# Construction of tissue-engineered vascular grafts with enhanced patency by integrating heparin, cell-adhesive peptide, and carbon monoxide nanogenerators into acellular blood vessels

**DOI:** 10.1016/j.bioactmat.2023.12.015

**Published:** 2023-12-28

**Authors:** Yonghong Fan, Juan Pei, Yinhua Qin, Huifang Du, Xiaohang Qu, Wenya Li, Boyue Huang, Ju Tan, Yong Liu, Gang Li, Ming Ke, Youqian Xu, Chuhong Zhu

**Affiliations:** a Department of Anatomy, Engineering Research Center for Organ Intelligent Biological Manufacturing of Chongqing, Key Lab for Biomechanics and Tissue Engineering of Chongqing, Third Military Medical University, Chongqing, 400038, China; b Laboratory of Basic Medicine, The General Hospital of Western Theater Command, Chengdu, 610083, China; c Engineering Research Center of Tissue and Organ Regeneration and Manufacturing, Ministry of Education, Chongqing, 400038, China; d State Key Laboratory of Trauma and Chemical Poisoning, Chongqing, 400038, China; e Department of Plastic and Aesthetic Surgery, Southwest Hospital, Third Military Medical University, Chongqing, 400038, China

**Keywords:** Tissue engineered vascular grafts, Carbon monoxide, Heparin, Endothelial-to-mesenchymal transition

## Abstract

Small-diameter tissue-engineered vascular grafts (sdTEVGs) have garnered significant attention as a potential treatment modality for vascular bypass grafting and replacement therapy. However, the intimal hyperplasia and thrombosis are two major complications that impair graft patency during transplantation. To address this issue, we fabricated the covalent-organic framework (COF)-based carbon monoxide (CO) nanogenerator-and co-immobilized with LXW-7 peptide and heparin to establish a multifunctional surface on TEVGs constructed from acellular blood vessels for preventing thrombosis and stenosis. The cell-adhesive peptide LXW-7 could capture endothelial-forming cells (EFCs) to promote endothelialization, while the antithrombotic molecule heparin prevented thrombus formation. The reactive oxygen species (ROS)-triggered CO release suppressed the adhesion and activation of macrophages, leading to the reduction of ROS and inflammatory factors. As a result, the endothelial-to-mesenchymal transition (EndMT) triggered by inflammation was restricted, facilitating the maintenance of the homeostasis of the neo-endothelium and preventing pathological remodeling in TEVGs. When transplanted *in vivo*, these vascular grafts exhibited negligible intimal hyperplasia and remained patent for 3 months. This achievement provided a novel approach for constructing antithrombotic and anti-hyperplastic TEVGs.

## Highlights

• Tissue-engineered vascular grafts have been constructed by integrating heparin, cell-adhesive peptide, and carbon monoxide nanogenerators into acellular blood vessels.
• The cell-adhesive peptide can capture endothelial-forming cells to promote endothelialization, while the heparin immobilization facilitates the inhibition of thrombus formation.
• Reactive oxygen species-triggered carbon monoxide release can eliminate endothelial-to-mesenchymal transition-induced pathological remodeling in the TEVGs by promoting inflammatory resolution.
• These multifunctional tissue-engineered vascular grafts have shown enhanced patency without thrombosis and severe intimal hyperplasia during implantation.

## Graphical abstract

**Figure:**
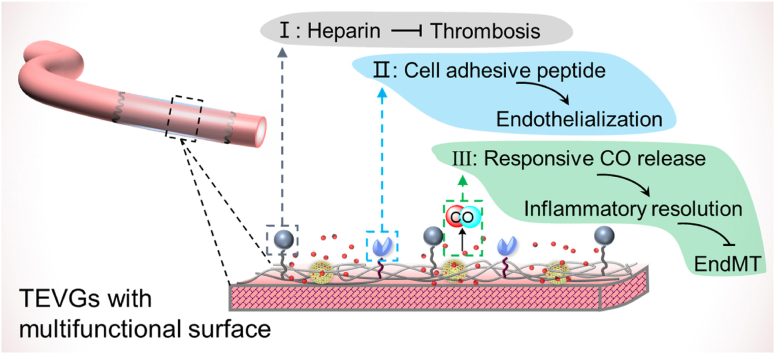

## 1 Introduction

Cardiovascular disease (CVD) is among the leading causes of death among humans globally. The defective blood vessels tend to interrupt blood supply to the surrounding tissues, resulting in the loss of organs or limbs in patients [1]. Although bypass grafting with autologous or synthetic vasculatures is often employed to restore blood flow, the risk of infection and thrombosis associated with synthetic materials, or the lack of suitable native vessels from patients, still urging us to seek feasible alternatives [2]. To date, TEVGs have shown promising prospects in treating CVDs or providing access to hemodialysis [3,4]. TEVGs can potentially mimic the properties of autografts, especially the mechanical properties and growth capacity [5]. Scientists have made significant efforts to create clinically viable TEVGs, but none of the products have succeeded commercially.

Acellular matrixes, generated from cultured cells or native tissues, provide a readily available option for constructing vascular grafts off the shelf [6]. They have favorable cytocompatibility and mechanical compliance, allowing for effective cell ingrowth and vascular remodeling. Ultimately, the TEVGs can be replaced by the host vascular tissue. However, thrombosis and intimal hyperplasia are still the two major complications that should be addressed in developing TEVGs with long-term patency [7,8]. It has been well acknowledged that endothelialization is critical for fabricating blood-contacting devices such as vascular grafts and stents [9]. Endothelial cells (ECs) lining in the natural vessel lumen plays a crucial role in maintaining vascular homeostasis, such as preventing thrombosis and stenosis by releasing regulatory molecules including nitric oxide (NO), heparin, and plasmin [10]. The absence of a functional endothelium on acellular TEVGs leads to a cascade of pathological reactions, including thrombogenesis, inflammation reactions, and smooth muscle cell (SMC) hyperplasia [11]. To address this, we have adopted various innovative approaches to simulate the functionality of native ECs and/or accelerate endothelialization on acellular TEVGs [[12], [13], [14]]. For instance, inspired by the phenomenon that ECs continuously generate endogenous antiplatelet substances via enzymatic reactions, we have developed a dual-enzyme biomimetic cascade to inhibit thrombosis by converting adenosine diphosphate (ADP) into adenosine monophosphate (AMP) and AMP into adenosine [15]. These strategies have been proved successful to some extent in improving the patency of TEVGs.

However, single-function design may be inadequate to achieve optimal outcomes in terms of simultaneously preventing thrombosis and intimal hyperplasia, as indicated by our previous study [16]. Although the TEVGs have been modified with bioactive molecules to render antithrombotic properties and prevent clot formation, the delayed generation of endothelium is insufficient to suppress the proliferation of SMCs and adherent circulating stem cells. Exposure to extracellular matrix components, especially collagen, tends to initiate the coagulation cascade before endothelialization occurs on the TEVGs [17]. In addition, the inflammatory responses induced by graft implantation play a crucial role in graft remodeling. Biochemical molecules released during inflammation can regulate the biological behavior of ECs and SMCs, influencing endothelialization and lumen patency of the vascular grafts [18]. Recent studies have identified inflammatory compounds, including ROS, TNF-α, IL-1β, and TGF-β, can act as triggers of EndMT [19,20]. During graft remodeling, the homing EFCs, such as ECs and endothelial progenitor cells (EPCs), can be activated by the inflammatory factors and undergo EndMT [21]. EndMT drives endothelial dysfunction, with EFCs losing their morphological and functional characteristics and subsequently causing thrombosis. These EFC-derived cells acquire biological properties similar to other cell lineages, such as fibroblasts, myofibroblasts, SMCs, and pericytes, leading to undesirable extracellular matrix deposition and intimal hyperplasia [22]. Moreover, these abnormal EFCs express numerous cell adhesion molecules and promote further inflammation [23]. Therefore, constructing multifunctional TEVGs which can prevent thrombosis, promote endothelialization and inflammatory resolution, and inhibit EndMT-mediated pathological remodeling is crucial to enhance graft patency.

**Scheme 1:**
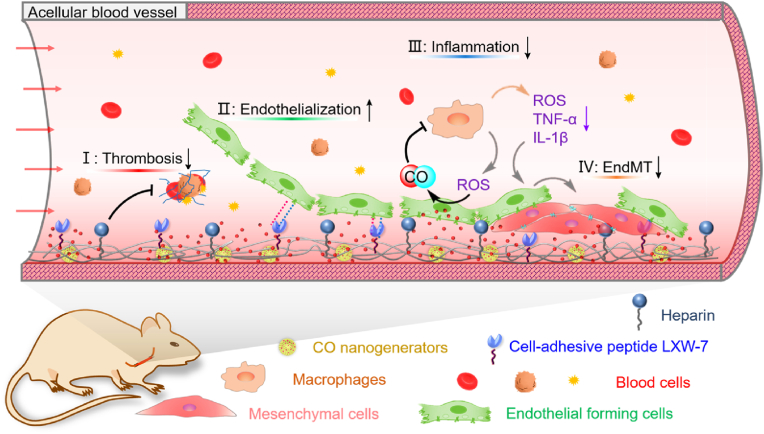
Schematic illustration of TEVG preparation by co-immobilization of heparin, cell-adhesive peptide, and carbon monoxide nanogenerators onto acellular blood vessels. These multifunctionalized TEVGs have demonstrated with enhanced patency by preventing thrombosis, capturing EFCs, inhibiting inflammation, and preventing EndMT.

In the present study, heparin, LXW-7 peptide and CO nanogenerators were integrated into the acellular blood vessels to serve as TEVGs for vascular replacement therapy. The amino groups in the acellular matrix provided binding sites for grafting heparin in the presence of carbodiimide (EDC) [24]. The covalent immobilization could maintain the stability and prolong the duration of heparin, imparting excellent antithrombotic properties to the TEVGs. This approach may avoid the side effects of systemic anticoagulant medications [25]. LXW-7 is a cyclic peptide with a high affinity for EPCs/ECs via αvβ3 integrin but low affinity for platelets and monocytes; immobilization of LXW-7 on biomaterial surfaces promotes endothelialization effectively [26]. Thanks to the presence of unnatural amino acids and their cyclic structure, LXW-7 exhibits significant stability against hydrolysis than the traditional linear peptides. In this study, we induced a diazido group at the peptide's terminal and immobilized LXW-7 onto the acellular matrix by a click chemistry method using dibenzocyclooctylene-N-hydroxysuccinimide ester (DBCO-NHS) as the linker. It is well known that CO can promote inflammatory resolution by reducing the production of pro-inflammatory cytokines, TNF-α, IL-1β, and MIP-1β, while enhancing the expression of anti-inflammatory factor IL-10 [[27], [28], [29]]. As a gasotransmitter, CO can be a promising therapeutic for modulating inflammation during TEVG implantation. Here, the CO nanogenerators, CO@COF, were synthesized by loading manganese carbonyl (MnCO) into COFs. And then these CO nanogenerators were anchored onto the TEVGs for responsive CO release. We hope these TEVGs can suppress thrombus formation, capture EPCs to induce endothelialization, and regulate the inflammatory response to prevent EndMT, thereby improving the patency of the TEVGs (Scheme 1). To validate our hypothesis, the *in vitro* CO release and biological functions of the CO@COF, the EPCs capture and the clot formation on the functional surface, the *in vivo* graft patency, the endothelialization, neointima formation, inflammatory response and EndMT in the TEVGs, were investigated here.

## 2 Materials and methods

### 2.1 Materials

1,3,5-Tris(4-aminophenyl)benzene (TPB) was purchased from Shanghai Tensus Biotech Co., Ltd., China. Hemoglobin from bovine blood (Hb), palladium chloride (PdCl_2_), N-Hydroxysuccinimide ester (NHS), 2-(N-morpholine) ethosulfonic acid (MES), 1-(3-Dimethyla minopropyl)-3-ethylcarbodiimide (EDC), and 1,4-Phthalaldehyde (PA) were purchased from Shanghai Macklin Biochemical CO., Ltd., China. Manganese carbonyl (MnCO) and Dibenzocyclooctyne-N-hydroxysuccinimidyl ester (DBCO-NHS) were obtained from Merck, USA. LXW-7 (cGRGDdvcK(N_3_-PEG_5_-CH_2_CH_2_COOH)–NH_2_) peptide was purchased from Shanghai ChinaPeptides Co., Ltd., China. Sodium dithionite (SDT) was purchased from Shanghai Aladdin Biotech Co., Ltd., China. Collagen type І from rat trails was from Corning, USA. Dihydroethidium (DHE) was obtained from ThermoFisher Scientific, USA. Lipopolysaccharide (LPS), heparin and TRITC-Phalloidin were obtained from Beijing Solarbio Life Sciences, China. 4′,6-diamidino-2-phenylindole (DAPI) and paraformaldehyde (PFA) were purchased from Wuhan Servicebio Co., Ltd., China. The RNase and DNase were purchased from Shanghai Yien Chemical Technology Co., Ltd., China. Dil-Ac-LDL was purchased from Shanghai Maokang Biotech Co., Ltd., China. Primary antibodies including TNF-α (ab205587, Abcam, USA), IL-1β (ab283818, Abcam, USA), iNOS (ab3523, Abcam, USA), Arginase 1 (sc-271430, Santa Cruz, USA), TGF-β (sc-52893, Santa Cruz, USA), CD144 (MAB9381, R&D system, USA), CD31 (ab24590, Abcam, USA), Vimentin (ab8978, Abcam, USA), α-SMA (bs-10196R, Bioss, China), the Alexa Fluor 488 and Alexa Fluor 594 conjugated secondary antibodies (Invitrogen, USA), the NO detection kit (S0021S, Beyotime, China), mouse TNF-α ELISA kit (KE10002, Proteintech, USA), mouse IL-1β ELISA kit (KE10003, Proteintech, USA), mouse TGF-β ELISA kit (KE10005, Proteintech, USA), mouse IL-10 ELISA kit (KE10008, Proteintech, USA), LIVE/DEAD staining kit (PF00007, Proteintech, USA), and CellMask™ (C10045, Invitrogen) were employed in this study. All chemicals and reagents were used as received.

### 2.2 Synthesis of COF nanoparticles

The COF nanoparticles were synthesized as described by Ma et al. with minor modifications [30]. Briefly, 0.04 mmol TPB and 0.06 mmol PA were dissolved in 5 mL of ethanol, respectively. The two solutions were mixed, and then 100 μL of acetic acid (12 M) was added immediately. After 24 h of stirring, the COF nanoparticles were collected by centrifugation (10,000 rpm), washed with ethanol, and dried under a high vacuum.

### 2.3 Preparation of CO nanogenerators

To prepare CO@COF, the COF nanoparticles (2 mg/mL) were dispersed in methanol, and then the MnCO (4 mg/mL) was dissolved into this suspension. After 5 min of sonication, the mixture was kept at room temperature under magnetic stirring for 24 h. Next, the mixture was evaporated under high vacuum until the volume decreased to about one-fifth of the total solvent. Finally, the CO@COF nanoparticles were collected by centrifugation and washed thrice with DI water. To evaluate the loading capacity (LC) and loading efficiency (LE) of COF, the supernatant was collected and the MnCO in the supernatant (*W*_1_) was detected using an ultraviolet–visible (UV–Vis) spectrophotometer (UNICO UV-4800, China). The LC and LE were calculated using the following formulas [31]:

$$LC\left(\%\right)=\frac{W_{0}-W_{1}}{W_{\text{COF}}}\times 100\%$$

$$LE\left(\%\right)=\frac{W_{0}-W_{1}}{W_{0}}\times 100\%$$

where *W*_COF_ is the amount of COF used and *W*_0_ is the amount of MnCO added. A standard curve was used to calculate the concentration of MnCO in the supernatant (Fig. S1).

### 2.4 Characterization

The morphology of the COF and CO@COF nanoparticles was observed using a scanning electron microscope (SEM, Hitachi SU8010, Japan) and a transmission electron microscope (TEM, JEOL-JEM 2100F, Japan). The size of the CO@COF sample in DI water was measured on a Malvern Zetasizer Nano S90 instrument (UK). Fourier transform–infrared spectroscopy (FT-IR) spectra were recorded on a ThermoFisher Nicolet IS5 spectrometer (USA). X-ray photoelectron spectroscopy (XPS) was performed using an x-ray photoelectron spectrometer (ThermoFisher, ESCALAB 250Xi, USA) with a monochromatic Al Kα x-ray source (*hυ* = 1486.6 eV). The Barret–Joyner–Halenda (BJH) method was employed to calculate the pore size distribution of the nano-COF based on the N_2_ adsorption–desorption isotherms, which were measured using a BET analyzer (BSD Instrument, BSD 3H–2000PS1, China).

SEM images of cells, platelets, and tissues were taken using ZEISS Crossbeam 340. The samples were fixed with 2.5 *v*/*v*% glutaraldehyde overnight and washed twice with 0.9 wt % normal saline. Subsequently, the samples were dehydrated with graded ethanol and *tert*-butanol. The samples were mounted onto SEM pucks using a conductive carbon tape, coated with gold for 45 s, and observed under SEM. Fluorescent images of cells were acquired under a confocal laser scanning microscope (CLSM, TCS SP8 DIVE, Leica Microsystems) or a fluorescence microscope (Leica, EVOS FL). For tissue slides, the fluorescent images were captured using an Olympus VS200 scanner. The acquisition settings remained constant to enable qualitative assessment of the fluorescent intensity. The mean fluorescent intensity (MFI) was measured using ImageJ software (National Institute of Health, Bethesda, MD, USA). The intensity was normalized to that of the unstained area to minimize the variability between images. The absorbance was determined using a microplate reader (Tecan, Infinite M200 pro). Quantitative flow cytometry was performed using a flow cytometer (BD Accuri C6 Plus, BD Biosciences).

### 2.5 MnCO release from CO@COF

To study the dynamics of MnCO release, 1 mg of CO@COF was dispersed in 300 μL of phosphate-buffered saline (PBS) (pH = 7.4) and continuously oscillated at 110 rpm and 37 °C. At the determined time point, the samples were centrifuged, and the supernatant was collected. An equivalent volume of fresh PBS was added. The release of MnCO was determined by measuring the concentration of manganese element in the supernatant using ICP-MS (PerkinElmer NexION 2000, USA).

### 2.6 Detection of CO release under inflammatory conditions

A typical procedure for monitoring CO release in response to ROS was performed as described by He et al. [32]. Briefly, Hb (5 μM) was first dissolved in PBS (pH = 7.4) and then 0.4 mg/mL SDT was added to reduce the Hb under a nitrogen atmosphere. After a 10-min reaction, 200 μg CO@COF was dispersed into 4 mL of the aforementioned Hb solution. Immediately, H_2_O_2_ (0, 50, 100, 200, 500 μM) was added, and the solution was sealed in a 4-mL UV quartz cuvette. At determined time point, the adsorption spectra of the solution (300–600 nm) were collected. The concentration of CO in the solution was calculated using the following formula:

$$C_{\text{CO}}=\frac{528.6\times I_{410\;\text{nm}}-304\times I_{430\;\text{nm}}}{216.5\times I_{410\;\text{nm}}+442.4\times I_{430\;\text{nm}}}C_{\text{Hb}}$$

A probe (COP-1) with green fluorescence was used to determine the CO release in the macrophages [33]. RAW264.7 macrophages were purchased from Chongqing Biospes Biotech Co., Ltd., China. The macrophages were cultured in a DMEM/high-glucose medium (Hyclone, USA) with 10 % fetal bovine serum (Hyclone). Cells that reached 80 % confluence were detached from the cell culture flask and dissociated into single-cell suspension mechanically. The cells were seeded at a density of 1 × 10^5^ cells/mL, and 12 h was left to allow cell attachment before LPS (200 ng/mL) and/or CO@COF (50 μg/mL) addition. After culturing for another 48 h, the cells were incubated with a CO probe (5 μM) and PdCl_2_ (5 μM) for 60 min, washed with PBS, and stained with CellMask (10 min, 1:1000 dilution).

To detect the ROS level in LPS-activated macrophages, the cells were seeded on 10-mm poly-l-lysine coated cell coverslips in a 24-well cell culture plate (1 mL, 5 × 10^4^ cells/mL). After 48-h incubation with LPS (200 ng/mL) and/or CO@COF (5 μg/mL), DHE (5 μM) was added to treat the cells for another 30 min at 37 °C in the dark. Immediately, the samples were taken out, washed with PBS, and observed under the fluorescence microscope.

For flow cytometry assay, the macrophages were seeded in a six-well cell culture plate (1 × 10^5^ cells/mL, 3 mL), cultured for 12 h, treated for 24 h by adding 50 μg/mL CO@COF on 0.4-μm Transwell, incubated with LPS (200 ng/mL) for another 24 h, and stained with CO probe or DHE.

### 2.7 Macrophage polarization and activation

Macrophage polarization, activation, and inflammatory cytokine secretion were measured by seeding cells on poly-lysine coated 24-well cell climbing coverslips (5 × 10^4^ cells/mL) and treating with LPS (200 ng/mL) and/or CO@COF (20 μg/mL) for 12 h after allowing them to attach for an initial 12-h period. Then, the cells were fixed with 4 % paraformaldehyde (PFA) for further immunofluorescence staining assay. The cell culture medium was collected, and the levels of TNF-α, IL-1, TGF-β, and IL-10 were measured using the corresponding enzyme-linked immunosorbent assay (ELISA) kits. NO secretion was determined with a NO detection kit. The experimental procedure was carried out in accordance with the instructions outlined in these kits.

### 2.8 ROS triggered cell death and EndMT in human umbilical vein endothelial cells

Human umbilical vein endothelial cells (HUVECs) were obtained from Chongqing Jiukang Medical Research Institute Co., Ltd. HUVECs (passage 5–10) were cultured in endothelial cell basal medium-2 (EBM-2, CC-3156; Lonza, USA) supplemented with EGM-2 BulletKit (CC-4176; Lonza). To evaluate the protective effect of CO release on ROS-induced apoptosis and EndMT, HUVECs were pretreated with CO@COF and then incubated with H_2_O_2_. Briefly, cells that achieved 80 % confluence were detached from the cell culture flask using 0.25 % trypsin and seeded on 10-mm poly-l-lysine-coated coverslips (900 μL, 4 × 10^4^ cells). After 12 h of culture, the cells were incubated with 50 μg CO@COF (100 μL on a 0.4-μm Transwell) for 24 h.

To determine the ROS level and cell apoptosis in the HUVECs, the cells were treated with 100 μM H_2_O_2_ for another 5 h and then stained with DHE or a LIVE/DEAD staining kit. Alternatively, the cells were stimulated with 50 μM H_2_O_2_ for 48 h, followed by immunofluorescence staining and RT-qPCR assay to evaluate ROS-induced EndMT in the HUVECs. The primer sequences used in this study are listed in Table S1.

### 2.9 EndMT in the presence of macrophages

To investigate whether inflammation-mediated EndMT could be suppressed by inhibiting the activation of inflammatory cells by responsive CO release, HUVECs were co-cultured with LPS-activated macrophages, and the function and phenotype switch of the ECs were evaluated [34]. Initially, macrophages (5 × 10^4^ cells) were seeded on Transwell with 3-μm pores and then treated with LPS (200 ng/mL) and CO@COF (20 μg/mL) for 24 h. The HUVECs were seeded on 24-well cell culture plates or 10-mm poly-l-lysine-coated coverslips. Then, the Transwell with macrophages were taken out and the cell culture medium was discarded, and put onto the HUVECs. After 48 h of incubation, the HUVECs were collected, and the F-actin, eNOS, CD144, CD31, vimentin, and α-SMA were stained and observed. RNA was extracted from the cells using a Total RNA Mini Kit from FavorPrep, and the relative expression levels of the EndMT-associated genes were measured using a qPCR analyzer (CXF96, Biorad, USA). To assess the uptake of Ac-LDL, the ECs were incubated with 10-μg Dil-Ac-LDL for an additional 4 h. The cells were carefully washed, stained with DAPI, and then observed under a fluorescent microscope.

### 2.10 Construction and characterization of TEVGs

All animal experiments in this study were performed following the approval from the Laboratory Animal Welfare and Ethics Committee of the Third Military Medical University (No. AMUWEC2020039). Male Sprague–Dawley rats with 200 g bodyweight were anesthetized, and their carotid arteries were harvested under sterile conditions. Subsequently, the blood vessels were rinsed with heparin solution, decellularized with SDS (0.075 %) for 4 h, and digested with RNase (0.1 %) and DNase (0.1 %) at 37 °C for 1 h. Next, the vessels were immersed in CO@COF (1 mg/mL) for 2 h. The previous step was repeated, and the weakly adsorbed nanoparticles were removed by washing with PBS. After incubation with a collagen solution (1 mg/mL) for 2 h, the vascular grafts were treated with DBCO-NHS solution (50 μM) and cross-linked with heparin (1 mg/mL) using EDC/NHS/MES. Finally, the LXW-7 peptide was immobilized by incubating the vascular grafts with azide-functionalized peptide (100 μM) for 4 h. TEVG with only heparin immobilization, TEVG with both heparin and LXW-7 peptide, and TEVG with heparin, LXW-7, and CO@COF were referred to as Control, LXW, and CO&LXW in this study. The TEVGs were examined by hematoxylin and eosin (HE) staining and SEM observation. A computer-controlled stretch test system from Reger Instruments (China) was used to determine the maximum load and tensile strength of the TEVGs.

### 2.11 Hemocompatibility and EPCs capture assessment

Fresh citrate-anticoagulant blood was obtained from an SD rat. A part of the blood was centrifuged at 1500 rpm for 15 min to separate the blood corpuscles, and the supernatant containing platelet-rich plasma (PRP) was collected. Platelet adhesion assay was carried out by incubating TEVGs with PRP at 37 °C for 45 min. The TEVGs were incubated with the whole blood at 37 °C for 45 min to evaluate thrombus formation. *In vivo* thrombogenicity was assessed by implanting the TEVGs to the left carotid arteries of SD rats for 24 h. Then, the samples were carefully rinsed with PBS and observed under SEM.

To assess the EPC-capturing ability of the LXW-7 peptide functionalized surface, the samples were prepared as follows: PLL-modified 10-mm-cell coverslips were coated with collagen, and LXW-7 peptide was grafted onto them via click reaction. The EPCs (CP-R134) were isolated from the peripheral blood of rats and cultured in a corresponding special medium (CM-R134). The cells and culture medium were both obtained from Procell Life Science & Technology Co., Ltd., China. EPCs that reached 80 % confluence were used. The samples were incubated with 500 μL of cells (5 × 10^4^ cells/mL) for 5 h under oscillation (60 rpm). After removing the nonadherent EPCs, the samples were visualized with TRITC-phalloidin.

### 2.12 *In vivo* implantation of TEVGs

Male SD rats weighing approximately 200 g were randomly divided into three groups. After being anesthetized, their left carotid arteries were exposed, and the TEVGs with 6 mm length were grafted using an anastomotic cuff technique [35]. Over the following weeks, the rats were fed with food and water provided *ad libitum*.

Doppler ultrasound assessment was performed after 2 weeks, 3 weeks, 4 weeks, and 3 months using an ultrasound imaging system (Esaote, Italy, or Vinno, China). Before ultrasonic evaluation, the rats were anesthetized and the hair around the neck was cleared. The color mode and pulse wave mode were used to evaluate the patency, diameter, and blood flow velocity of the TEVG. Computed tomography (CT) images after 3 months were captured on a small-animal CT scanning instrument (Bruker micro-CT, USA). Iohexol was used as the vascular contrast agent and injected into the left ventricle before measurement.

For histological and immunofluorescence staining, the TEVGs were fixed with 4 % PFA, dehydrated with 30 % sucrose, embedded in paraffin or O.C.T. Compound, and finally sectioned into 5 μm/10 μm thick slices mounted on glass slides. Subsequently, these tissue sections were dewaxed using xylene and rehydrated in an alcohol gradient. They were then subjected to HE and immunofluorescence staining. To evaluate the endothelial growth within the TEVG lumen, the vessels were longitudinally split and observed under an SEM.

### 2.13 Immunofluorescence staining assay for cells and tissues

Before staining, the paraffin sections from the TEVG were dewaxed and hydrated. Antigen retrieval was performed using a citric acid buffer. Cells for immunofluorescence staining were seeded on polylysine-coated coverslips and fixed with 4 % PFA for 15 min after stimulation. The tissue sections or cells were washed with PBS and then treated with 0.1 % Triton X-100 and 3 % BSA successively. Subsequently, the samples were incubated with primary antibody at 4 °C in a humid chamber overnight and visualized with Alexa Fluor 488– or Alexa Fluor 594–conjugated secondary antibody after washing with PBS. In this study, all primary antibodies were used at a 1:200 dilution and the secondary antibodies at a 1:1000 dilution. TRITC-phalloidin (200 nM) was used to visualize the F-actin for morphological assessment of the cells. Prior to imaging, the cell nuclei were stained with DAPI (2 μg/mL) at room temperature for 15 min.

### 2.14 Statistical analysis

All data are shown as mean ± standard deviation (SD) based on minimum three replicates. GraphPad Prism 7 software was used to generate charts and perform statistical analysis. Comparisons between two groups were performed by using the two-tailed Student's unpaired *t*-test or one-way analysis of variance (ANOVA) followed by Tukey correction. A P < 0.05 was considered statistically significant and the asterisks represent different levels of significance (*p < 0.05, **p < 0.01, ***p < 0.001), *ns* means there was no significant difference (p > 0.05).

## 3 Results

### 3.1 Preparation and characterization of COF-based CO nanogenerators

**Fig. 1:**
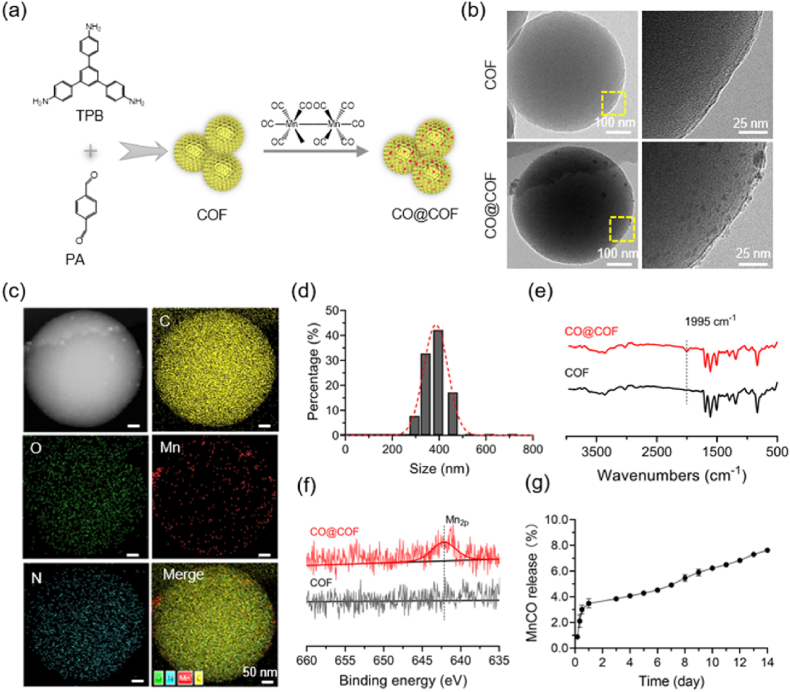
Synthesis and characterization of the COF-based CO nanogenerators. (a) Schematic diagram of the synthesis of CO nanogenerators. (b) TEM images of COF and CO@COF. (c) Elemental mapping of C, O, Mn, and N in the CO@COF. (d) Size distribution of CO@COF in water determined by dynamic light scattering (DLS) measurements. (e) FT-IR spectra of COF and CO@COF. (f) High-resolution XPS spectra of Mn_2p_ in COF and CO@COF. (g) Release profile of MnCO from the CO@COF.

COFs represent a new class of polymers with extremely high porosity and can be ideal candidates for drug delivery. In this study, the COF nanoparticles were synthesized at room temperature using TPB and PA as building blocks and then loaded with MnCO to prepare CO nanogenerators (Fig. 1a). XRD pattern showed that the resultant COF nanoparticles had poor crystallinity, which might be attributed to the solvent used in preparing this material (Fig. S2) [30]. BJH analysis of the COFs validated a narrow pore width distribution centered at 2.72 nm, and the pore size was much larger than the MnCO molecule, indicating that this material might have significant LC for this CO prodrug (Fig. S3). The EE and LC of the nano-COFs reached up to (23.6 ± 0.7) % and (47.2 ± 1.4) %, respectively. SEM and TEM images revealed that the resulting COF nanoparticles exhibited uniform spherical morphology and smooth surface (Fig. 1b and Fig. S4). The incorporation of MnCO did not significantly influence the morphology of the COFs, except that some drug crystals were observed in the CO@COF. Elemental mapping of the CO@COF confirmed the uniform distribution of manganese in a granular form in the nano-COFs (Fig. 1c). The size of the CO nanogenerators in deionized water was measured, and the result showed that the diameter was 381.2 ± 47.0 nm (Fig. 1d). The appropriate diameter allows for easy immobilization of these CO nanogenerators onto the decellularized vascular matrix. FT-IR and XPS analyses were performed to further confirm the successful loading of MnCO. As shown in Fig. 1e, a new peak at 1995 cm^−1^ emerged in the FT-IR spectrum of CO@COF, which could be attributed to the absorption of MnCO [36]. Also, the high-resolution spectra of XPS showed that an obvious Mn_2p_ peak appeared in CO@COF (Fig. 1f). The CO@COF had extremely slow MnCO release due to its strong hydrophobicity, making these CO nanogenerators capable of long-term function (Fig. 1g).

### 3.2 Responsive CO release and its inhibitory effects on the polarization and activation of macrophages

**Fig. 2:**
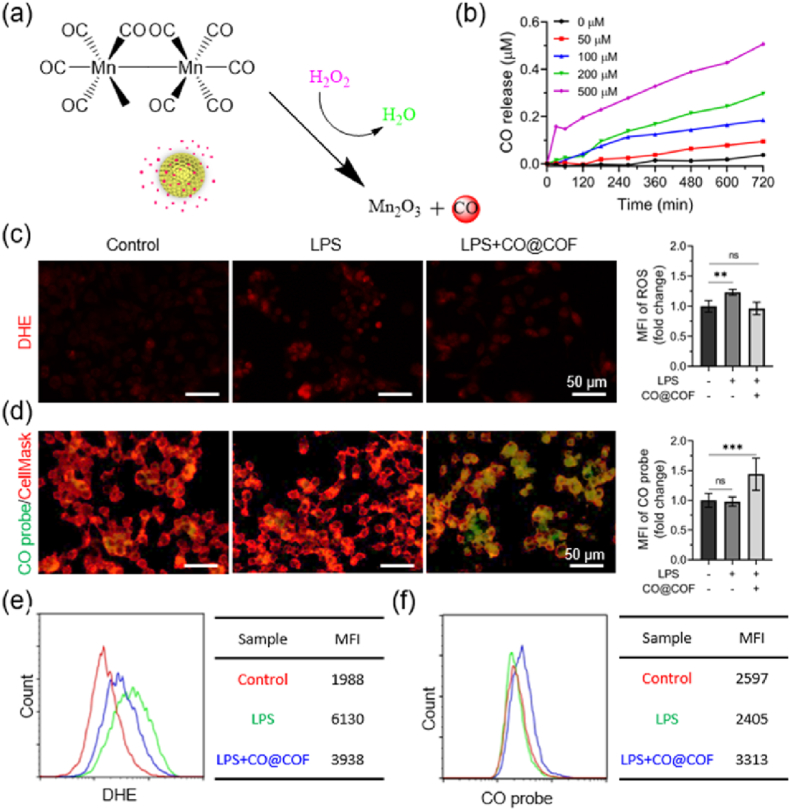
Responsive release of CO triggered by ROS. (a) Schematic diagram of CO generation by splitting MnCO in the presence of H_2_O_2_. (b) Cumulative CO release from CO@COF under various concentrations of H_2_O_2_. (c) ROS level as visualized by DHE. (d) Fluorescent imaging of the RAW264.7 cells where a CO probe was used to detect the CO release. (e) Flow cytometry analysis of ROS generation and (f) CO release in the RAW264.7 cells. Control, cells without any treatment; LPS, cells incubated with LPS (200 ng/mL) for 48 h; LPS + CO@COF, cells incubated with LPS (200 ng/mL) and CO nanogenerators (5 μg/mL) for 48 h. Data are presented as means ± SD (n ≥ 3) and analyzed using one–way ANOVA, *p < 0.05, **p < 0.01, ***p < 0.001, *ns*: not significant.

In the inflammatory microenvironment, the cells such as monocytes and macrophages secrete a large amount of ROS, including H_2_O_2_, superoxide anion (O_2_^•-^), and hydroxyl radical (•OH) [37]. Among these components, H_2_O_2_ can be decomposed into •OH through a Fenton-like reaction catalyzed by the central Mn ion. Then, the •OH further oxidizes and competitively coordinates with the central Mn in MnCO, causing the release of CO (Fig. 2a) [36]. Meanwhile, the H_2_O_2_ and •OH are eliminated along with the cleavage of MnCO. To examine the responsive CO release profile of the CO@COF, H_2_O_2_ at different concentrations was added to PBS (pH = 7.4) to simulate the ROS-rich inflammatory environment. The CO release was monitored using hemoglobin (Fig. S5a). The UV–Vis spectra under 500 μM H_2_O_2_ showed a perceptible decrease in absorbance at 557 nm, while the peak at 430 nm decreased and shifted to 410 nm (Fig. S5b). This implied a transition from Hb to HbCO, indicating that the CO@COF could respond to H_2_O_2_ to produce CO over time. Fig. 2b demonstrates that the rate of CO release depended on reaction time and H_2_O_2_ concentration, with CO release occurring slowly at low H_2_O_2_ concentrations (<100 μM).

The CO probe is an extremely useful tool in detecting CO within living cells. In this study, we monitored the responsive release of CO in macrophages using a specific CO probe known as COP-1 (Fig. S6). Given that the CO release from the nanogenerators is ROS-dependent, we first assessed the level of ROS in mouse macrophages following LPS treatment, as LPS serves as a potent activator for macrophages and can induce ROS secretion [38]. As visualized by DHE, ROS production increased remarkably in the LPS-activated macrophages, whereas it considerably decreased in the presence of CO@COF (Fig. 2c). From Fig. 2d, no significant green fluorescence was observed in the cells in the Control group and LPS groups after incubation with the CO probe and PdCl_2_ for 60 min. However, when the CO nanogenerators were added to the LPS-activated macrophages and incubated for 48 h, strong green fluorescence appeared. The fluorescence changes, as indicated by DHE (Fig. 2e) and COP-1 (Fig. 2f) staining, were further confirmed through flow cytometry analysis. These results indicated that CO was released in the inflammation environment with a high ROS content, and the ROS were incidentally removed from the inflammatory sites [39]. Uncontrolled release or high concentration of CO can cause obvious side effects, whereas the responsive CO release of our CO nanogenerators has the potential to significantly enhance therapeutic efficacy and mitigate the risk of CO poisoning [40].

**Fig. 3:**
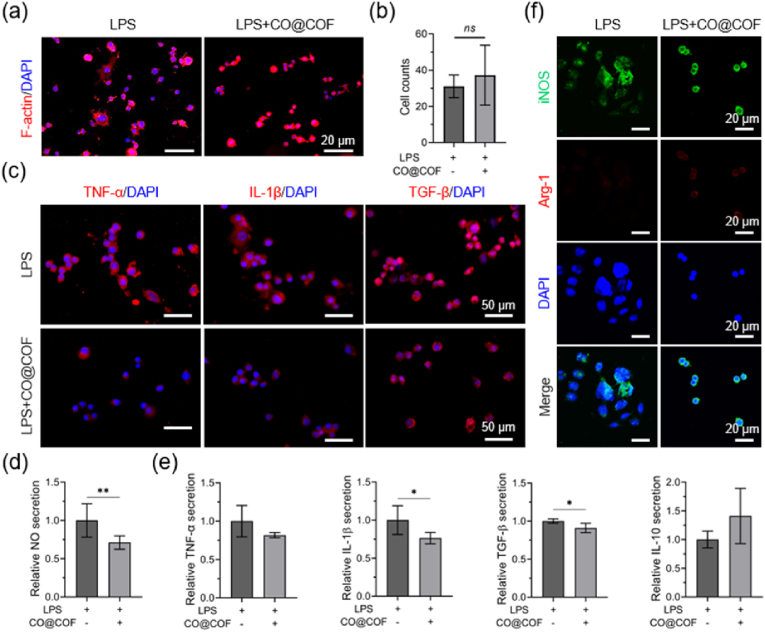
Responsive CO release inhibited the activation of macrophages. (a) Representative images of the adherent RAW264.7 cells, which were stained with TRITC-phalloidin and DAPI. (b) Cell counts of the macrophages on the cell climbing coverslips. (c) Immunofluorescent staining of TNF-α, IL-1β, and TGF-β in the macrophages. (d) Relative NO secretion of the adherent macrophages. (e) Relative TNF-α, IL-1β, TGF-β, and IL-10 secretion detected using ELISA kits. (f) Immunofluorescence staining of iNOS/Arg-1 in the adherent RAW 264.7 cells; the cell nuclei were strained with DAPI. Data are presented as means ± SD (n ≥ 3) and analyzed using two-tailed Student's unpaired *t*-test, *p < 0.05, **p < 0.01, ***p < 0.001, *ns*: not significant.

The anti-inflammatory effects of the CO@COF were determined in the LPS-activated macrophages. As shown in Fig. 3a, the adherent LPS-activated cells presented a rounded morphology when exposed to CO@COF, whereas many cells were deformed and their parapodium stretched out in the LPS group. The spread and extension of the pseudopodia indicated the polarization and activation of the macrophages, making them more likely to secrete inflammatory factors [41]. Moreover, a significant reduction in cellular counts was observed within the CO@COF group (Fig. 3b). The expression of TNF-α, IL-1β, and TGF-β in the adherent macrophages was evaluated by immunofluorescence staining. As shown in Fig. 3c, all of these inflammatory factors were highly expressed in the LPS-activated cells, while the addition of CO@COF inhibited their expression, especially TNF-α and IL-1β. The NO, TNF-α, IL-1β, TGF-β, and IL-10 in the cell culture medium were determined using corresponding kits. The results indicated that the presence of CO@COF downregulated the secretion of NO, TNF-α, IL-1β, and TGF-β, but increased the secretion of IL-10 (Fig. 3d and e). Furthermore, phenotypic analysis was performed by staining Arg-1 and iNOS in the macrophages. The fluorescent images showed that the LPS-activated cells expressed high iNOS but extremely weak Arg-1. In contrast, cells exposed to CO@COF exhibited increased expression of Arg-1, suggesting that the cells change its phenotype from pro-inflammatory M1 to anti-inflammatory M2 (Fig. 3f). It has been reported that moderate CO release can inhibit the activation of the macrophages, downregulate the expression of TNF-α and IL-1β, and promote the secretion of IL-10 [42]. Further, the inhalation of low-dose CO gas (250 ppm) led to a marked reduction in the number of M1 macrophages, but an increase in the proportion of M2 macrophages [43]. Hence, these results suggested that the CO@COF nanoparticles were capable of promoting inflammatory resolution through the responsive CO release.

### 3.3 CO release prevented inflammation-mediated injury and EndMT in ECs

**Fig. 4:**
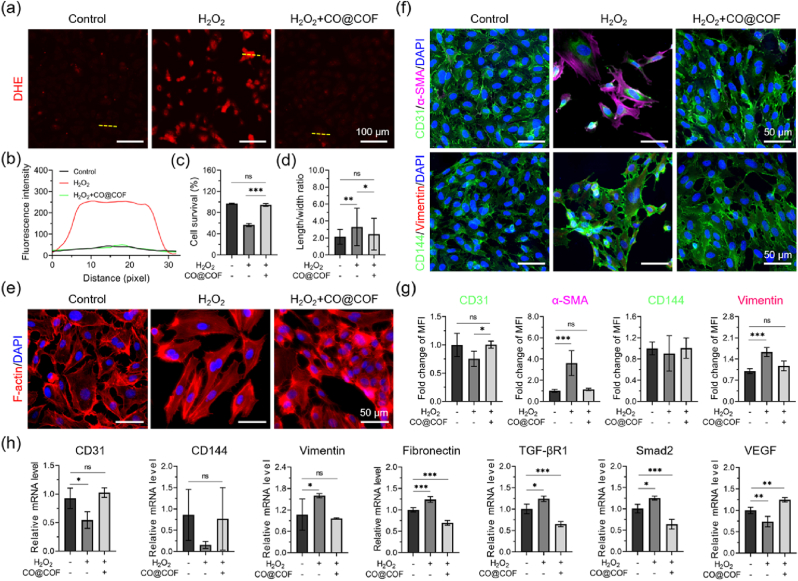
Responsive CO release protected HUVECs from ROS-mediated apoptosis and EndMT. (a) ROS level in HUVECs as visualized by DHE; the cells were pretreated with 50 μg/mL CO@COF for 24 h and then cultured with 100 μM H_2_O_2_ for 5 h. (b) Fluorescence intensity of the DHE-stained cells (designated with a yellow line). (c) Viability of the HUVECs plotted by analysis of the AM/PI staining images. (d) Statistical analysis of the length-to-width ratio of HUVECs. (e) Morphological changes in HUVECs; the cells were stained with TRITC-phalloidin and DAPI. (f) Immunofluorescence staining of the biomarker CD31 (green), α-SMA (purple), CD144 (green), vimentin (red) of the cells, the cell nuclei were stained with DAPI, the HUVECs were first pretreated with 50 μg/mL CO@COF for 24 h and then exposed to 50 μM H_2_O_2_ for 48 h. (g) Corresponding fold change in MFI of the CD31, α-SMA, CD144, and vimentin. (h) Relative mRNA expression of CD31, CD144, vimentin, fibronectin, TGF-βR1, Smad2, and VEGF detected by qPCR in HUVECs. Data are presented as means ± SD (n ≥ 3) and analyzed using one–way ANOVA, *p < 0.05, **p < 0.01, ***p < 0.001, *ns*: not significant.

Inflammatory responses are inevitable during the implantation of TEVGs and play a key role in influencing the patency of vascular grafts. Inflammatory cells produce numerous regulators, some of which can lead to EC dysfunction and trigger EndMT [44]. We expected to maintain the biological function of the neo-endothelium by immobilizing the prepared CO nanogenerators onto the TEVGs. To verify our assumption, exogenous H_2_O_2_ was added into the culture medium of HUVECs to investigate the protective effects of CO@COF against ROS-mediated apoptosis and EndMT. After treating HUVECs with 100 μM H_2_O_2_ for 5 h, the intracellular ROS level significantly increased as visualized by DHE. However, this increase in ROS was effectively reduced in cells pretreated with CO nanogenerators (Fig. 4a and b). Exposure to such a high concentration of H_2_O_2_ resulted in severe apoptosis in the ECs, with less than 60 % of the cells surviving compared with that in the Control group. Notably, the survival rate of the HUVECs pretreated with CO@COF was comparable to that in the Control group, indicating that CO release could protect cells from ROS-induced apoptosis (Fig. 4c and Fig. S7) [42]. Prolonged exposure to H_2_O_2_ would not only cause cell injury but also lead to EndMT. Therefore, the phenotype change of the HUVECs was evaluated by incubating the cells with 50 μM H_2_O_2_ for 48 h. Obviously, the length-to-width ratio of the ECs increased markedly in the presence of H_2_O_2_ (Fig. 4d). The ECs, which were originally arranged like paving stones, became disorganized and presented an elongated spindle shape (Fig. 4e). However, cells pretreated with CO nanogenerators showed limited changes in morphology compared with that of the natural HUVECs. Immunofluorescent staining was employed to assess the change in the biomarkers associated with EndMT in the HUVECs (Fig. 4f). The endothelial markers, CD31 and CD144, were highly expressed in the native ECs, whereas no obvious expression of α-SMA and vimentin was observed. After incubation with H_2_O_2_, the cells expressed α-SMA and vimentin. In contrast, the expression of these biomarkers in the cells pretreated with CO@COF showed very little difference from that in the normal ECs (Fig. 4g). RT-qPCR analysis showed that the expression of genes encoding CD31, CD144, and VEGF decreased upon H_2_O_2_ stimulation, but the cells pretreated with CO@COF restored the expression of these genes. In addition, the genes encoding vimentin, TGF-βR1, fibronectin, and smad2 were upregulated by H_2_O_2_, whereas the expression of these genes was suppressed in the cells pretreated with CO nanogenerators (Fig. 4h).

The co-expression of both EC markers (CD31 and CD144) and mesenchymal cell markers (α-SMA, vimentin, and fibronectin) is a typical characteristic of EndMT [45]. The TGF-βR1 (also known as ALK-5) associated signaling pathway, executed through the phosphorylation of Smad-2/3, plays a key role in TGF-β-mediated EndMT [46,47]. Studies have found that genes encoding TGF-β can be upregulated by ROS [48]. Exposure to ROS leads to the secretion of TGF-β1 and TGF-β2 in ECs, leading to EndMT through the activation of ALK-5 [49]. In addition, ROS can mediate the expression of TGF-β1 and *p*-Smad2 in cardiomyocytes [50]. Low doses of H_2_O_2_ (0.5–10 μM) are conducive to VEGF production in ECs, which in turn protects cells from apoptosis. However, the exposure of ECs to high concentrations of H_2_O_2_ results in severe cell injury [51]. The CO release has been identified to inhibit the expression of ALK-5 and *p*-Smad2, thereby reducing sensitivity to TGF-β1 [52,53]. Moreover, CO can promote the secretion of VEGF from ECs and the surrounding cells such as VSMCs, macrophages, and astrocytes, and then suppress EndMT through the VEGFR2-associated signaling pathway [22,54,55]. In this experiment, HUVECs were incubated with 50 or 100 μM H_2_O_2_, resulting in cell death and EndMT. However, the addition of CO nanogenerators prevented H_2_O_2_-induced apoptosis and EndMT. The possible mechanism by which CO prevents ROS-mediated cell death and EndMT involves downregulating Smad2 and TGF-βR1 expression, promoting VEGF secretion, and consuming ROS in ECs. The changes observed in ROS levels, and protein and gene expression supported our assumptions.

**Fig. 5:**
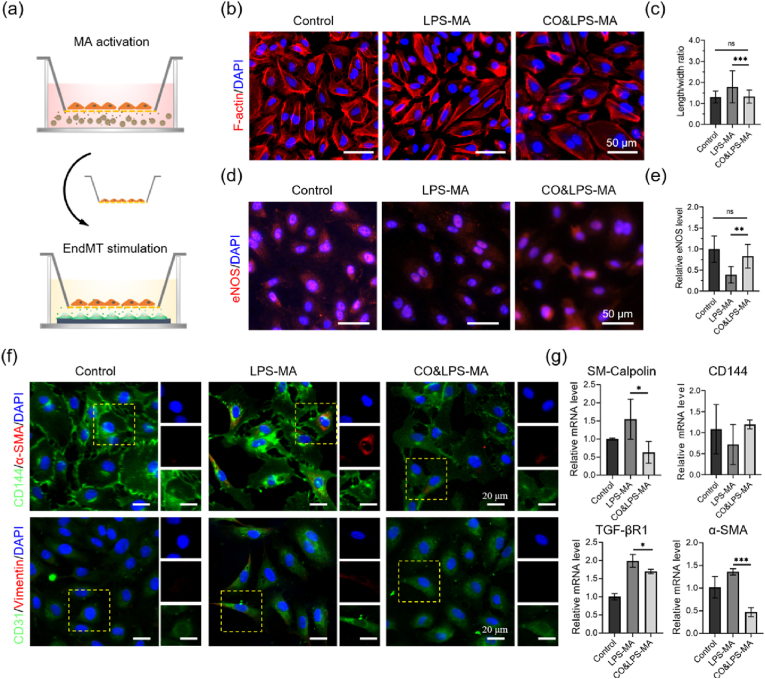
CO release prevented inflammatory-induced EndMT by inhibiting macrophage activation. (a) Schematic diagram of cell stimulation: macrophages were seeded on a Transwell with 3-μm pores and treated with LPS (200 ng/mL) and CO@COF (20 μg/mL) for 24 h, after which the macrophages were co-cultured with HUVECs for another 48 h. (b) Morphological changes of the HUVECs: cells were stained with TRITC-phalloidin and DAPI. (c) Statistical analysis of the cell length-to-width ratio of the HUVECs. (d) Immunofluorescence staining of eNOS (red), with cell nuclei stained with DAPI. (e) Corresponding relative eNOS expression plotted by analyzing the mean fluorescence intensity. (f) Expression of EndMT-associated markers in the HUVECs: CD31 and CD144 were stained green, α-SMA and vimentin were stained red, and the nuclei were stained with DAPI. (g) Quantitative analysis of mRNA for SM-calponin, CD144, TGF-βR1, and α-SMA by RT-qPCR. Data are presented as means ± SD (n ≥ 3) and analyzed using one–way ANOVA, *p < 0.05, **p < 0.01, ***p < 0.001, *ns*: not significant.

Previously, the CO@COF was demonstrated to inhibit the activation of macrophages and reduce the secretion of pro-inflammatory cytokines. Here, the HUVECs were incubated with macrophages to assess the efficacy of CO nanogenerators in suppressing EndMT by promoting inflammation resolution. First, the macrophages were treated with LPS and CO@COF for 24 h on a Transwell. Then, the macrophages were transferred onto the HUVECs and co-cultured for another 48 h (Fig. 5a). Upon the stimulation of LPS-activated macrophages, the shape of the HUVECs changed from a paving stone to a spindle (Fig. 5b). Also the length-to-width ratio of these ECs was higher than that in the Control group (Fig. 5c). In contrast, the macrophages treated with both LPS and CO@COF had very little influence on the morphology and length-to-width ratio of the HUVECs. Immunofluorescence staining of the intracellular eNOS revealed that the HUVECs exposed to LPS-activated macrophages downregulated this synthase. In contrast, the ECs co-cultured with macrophages treated with LPS and CO@COF displayed no discernible difference in eNOS expression compared with that in the Control group (Fig. 5d and e). Meanwhile, the Ac-LDL uptake of some ECs was attenuated by the LPS-activated macrophages to a certain extent (Fig. S8). However, the Ac-LDL uptake was not significantly impacted by the LPS treatment or the presence of CO@COF-treated macrophages. As shown in Fig. 5f, the ECs treated with LPS-activated macrophages expressed not only CD31 and CD144 but also α-SMA and vimentin. The cells co-cultured with LPS and CO@COF-treated macrophages, in contrast, had a negligible difference in expressing these biomarkers compared with the native ECs. The expression of the gene encoding CD144 in ECs was reduced by LPS-activated macrophages, but the LPS and CO@COF-treated macrophages did not influence the expression of this gene (Fig. 5g). Furthermore, incubation with LPS-activated macrophages resulted in the upregulation of genes encoding α-SMA, SM-calponin, and TGF-βR1 in ECs. In contrast, ECs co-cultured with LPS and CO@COF-pretreated macrophages had relatively lower expression levels of these genes.

The infiltration and activation of inflammatory cells cause acute or chronic inflammation during implantation, which mediates the pathological remodeling in TEVGs. Inflammation-induced EndMT may result in complications such as vascular stenosis, calcification, fibrosis, and thrombosis. In this experiment, the changes in morphology, biomarkers, and genes indicated that the activation of macrophages significantly affected the function of ECs by promoting EndMT. In contrast, the CO release can inhibit the activation of macrophages and reduce the secretion of inflammatory cytokines, thereby preventing EndMT. Hence, the CO nanogenerators were expected to enhance the patency of TEVGs by promoting inflammation resolution, inhibiting the EndMT in the homing EFCs and maintaining its biological functions.

### 3.4 Construction, EPCs capture and thrombus formation of the TEVGs

**Fig. 6:**
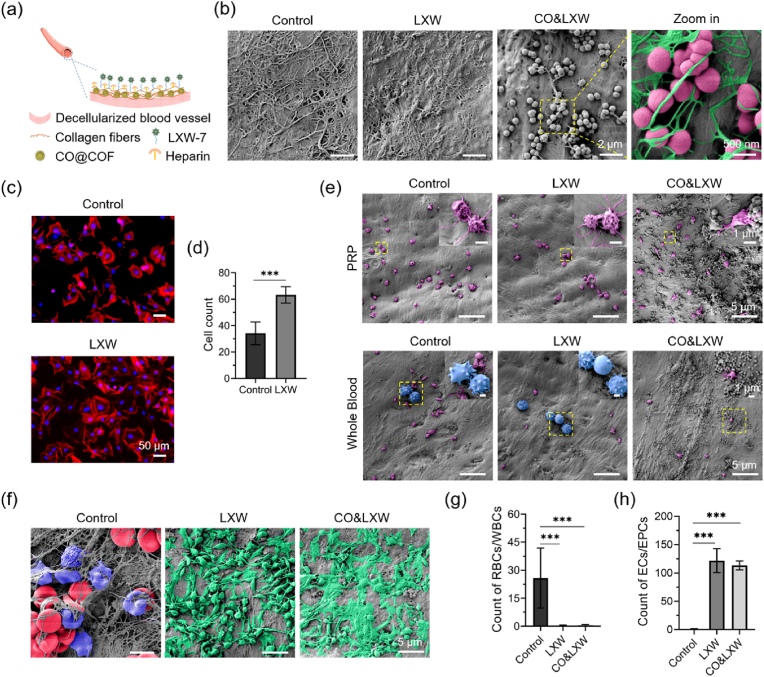
Preparation, EPC capture, and thrombogenicity of the TEVGs. (a) Schematic illustration of the construction of TEVGs by combining heparin, cell-adhesive peptide LXW-7, and CO nanogenerators. (b) Representative photographs of the TEVGs under SEM observation. The false-colored SEM image shows the CO nanogenerators (carmine) anchored by the collagen fibers (green). (c) Captured EPCs on the LXW-7 functionalized surface. The cells were stained with TRITC-phalloidin and DAPI. (d) Analysis of cell count of the adherent EPCs. (e) False-colored SEM images of blood cell adhesion on the TEVGs after incubation with whole blood and PRP for 45 min. The WBCs were colored blue, and the platelets were colored purple. (f) False-colored SEM images of TEVGs after implantation for 24 h. The WBCs, RBCs, and ECs/EPCs were colored blue, red, and green, respectively. (g) Cell count of the adherent WBCs and RBCs on the TEVGs as measured from the SEM images. (h) Statistical analysis of cell count of the captured ECs/EPCs as measured from the SEM images. Data are presented as means ± SD (n ≥ 3) and analyzed using one–way ANOVA, *p < 0.05, **p < 0.01, ***p < 0.001, *ns*: not significant.

The TEVGs were constructed by integrating the CO nanogenerators, heparin, and LXW-7 peptide onto the acellular blood vessels (Fig. 6a). Many aldehyde groups remained on the surface of the hydrophobic CO@COF, making these CO nanogenerators easily immobilized on the decellularized vascular matrix through hydrophobic interactions or the formation of amide bonds. Collagen was employed to trap the CO@COF, and then the heparin was immobilized using an EDC/NHS/MES system. The DBCO-NHS linker was introduced to bind LXW-7 peptide through bioorthogonal reaction. This method for LXW-7 grafting was mild in reaction conditions, fast in reaction rate, and highly specific, and did not have any significant effect on the bioactivity of this peptide. Decellularization resulted in a decline in the mechanical properties, as evidenced by the reduced tensile strength. However, immobilizing these functional components enabled the TEVGs to exhibit strength comparable to that of natural blood vessels (Fig. S9). Notably, the color of the vascular graft changed from white to yellow due to the presence of CO@COF (Fig. S10). The HE staining of the TEVGs revealed that all the cells were completely removed, and the tubular structure of the blood vessel remained (Fig. S11). In addition, many CO@COF nanoparticles were observed on both the luminal and external surfaces of the TEVGs. As shown in Fig. 6b, no significant difference in the luminal surface structure was found to exist between the LXW group with LXW-7 immobilization and the Control group with only heparin grafting. However, in the CO&LXW group, in which the cells were decorated with heparin, LXW-7, and CO@COF, the CO nanogenerators were widely distributed on the luminal surface. Moreover, these nanoparticles were firmly tied up by the collagen fibers, enabling the CO@COF to resist the scour of blood flow. Next, the EPC capture and thrombus formation of the TEVGs were evaluated. The results showed that the LXW-7-immobilized surface effectively enhanced the capture of EPCs under dynamic culture (Fig. 6c and d). After incubation with PRP and whole blood, only a few platelets adhered and spread on the TEVGs (Fig. 6e). Particularly, many white blood cells (WBCs) were found on the TEVGs except in the CO&LXW group. After being implanted into the carotid artery of SD rats for 2 h, many WBCs and red blood cells (RBCs) were entrapped on the heparin-immobilized TEVGs (Fig. 6f and g). It is well known that the WBCs, including granulocytes, monocytes, and lymphocytes, can secrete various cytokines such as chemotactic factors, interleukins, interferons, tumor necrosis factors, and initiate the inflammatory and immunologic cascade. In contrast, the luminal surface of TEVGs in the LXW and CO&LXW groups was covered with endothelioid cells (Fig. 6h). We speculated that these endothelioid cells were the circulating ECs and EPCs captured by the LXW-7 peptide. The mobilization and recruitment of the EFCs, especially the EPCs, have been demonstrated to be crucial for rapid endothelialization in TEVGs. Thus, our multifunctional TEVGs with the immobilization of heparin, LXW-7, and CO@COF possessed significant potential to maintain patency for a long time *in vivo*.

### 3.5 Endothelialization and neointimal hyperplasia of the TEVGs in an SD rat model

**Fig. 7:**
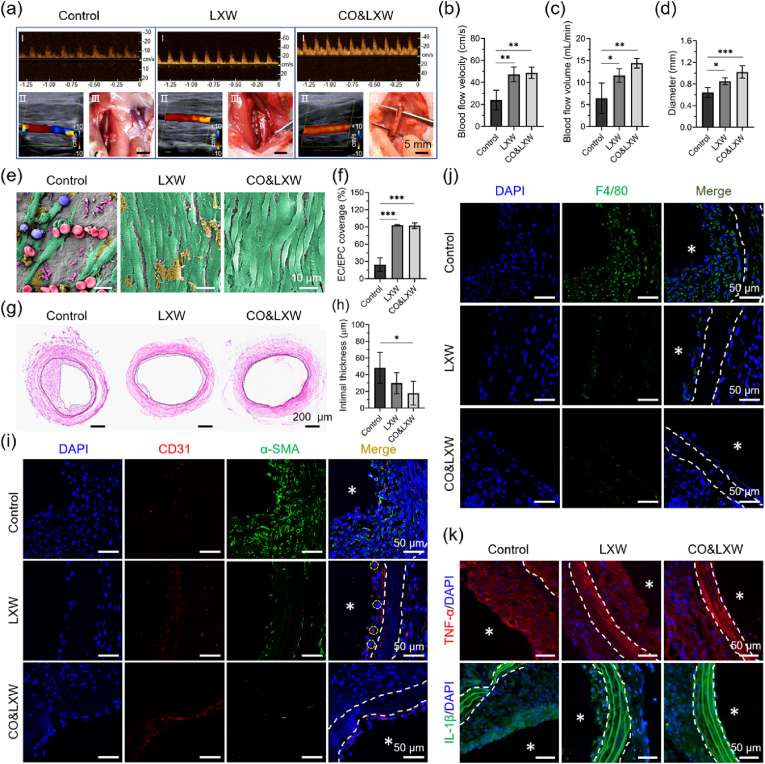
*In vivo* implantation of the TEVGs for 14 days in the carotid artery of SD rats. (a) Macroscopic views of ultrasound images and TEVG implantation. Statistical analysis of (b) blood flow velocity, (c) blood flow volume, and (d) lumen diameter of the TEVGs. (e) SEM observation of the vascular grafts, the images were false-colored, the WBCs, RBCs, platelets, ECs/EPCs, and the adhered plasma proteins are stained blue, rose, purple, green, and yellow, respectively. (f) Coverage ratio of the EPCs/ECs in the lumen of the vascular grafts. (g) Representative HE images of the TEVGs. (h) Statistical analysis of the neointimal thickness. (i) Immunofluorescence staining of the biomarker CD31 (red) and α-SMA (green) in the TEVGs, with CD31^+^α-SMA^+^ cells marked with white circles. (j) Identification of macrophage infiltration using F4/80 staining. (k) Immunofluorescence staining of TNF-α (red) and IL-1β (green) in the TEVGs. Cell nuclei were stained with DAPI, and the lumen of the TEVGs was indicated with asterisks. Data are presented as means ± SD (n ≥ 3) and analyzed using one–way ANOVA, *p < 0.05, **p < 0.01, ***p < 0.001, *ns*: not significant.

Finally, the TEVGs were implanted into the left carotid arteries of the SD rats to verify their patency *in vivo* [56]. After 14 days post-surgery, Doppler ultrasound analysis was conducted, and the result revealed that all the TEVGs in the three groups remained patent. However, the ultrasound charts plotted under pulse wave mode and the color Doppler flow imaging displayed an irregular profile and a disturbed blood flow in the Control group, suggesting that the intimal hyperplasia occurred in these vascular grafts (Fig. 7a). Moreover, the filling degree of the TEVGs in the Control group was lower compared with that in the LXW and CO&LXW groups. The blood flow velocity in the Control group was measured at 23.9 ± 8.9 cm/s, while it increased to 47.3 ± 6.6 cm/s and 48.7 ± 5.2 cm/s in the LXW and CO&LXW groups, respectively (Fig. 7b). Meanwhile, the blood flow volume in the three groups was measured as 6.4 ± 3.4 mL/min, 11.6 ± 1.6 mL/min, and 14.4 ± 1.1 mL/min (Fig. 7c). The lumen diameter of the TEVGs in the Control group was 0.64 ± 0.01 mm, which was much smaller than that in the LXW group (0.85 ± 0.07 mm) and CO&LXW group (1.02 ± 0.12 mm) (Fig. 7d). Then, the TEVGs were collected and observed under an SEM. As shown in Fig. 7e, a large number of RBCs, WBCs, and platelets, but very few ECs, were found to adhere to the vascular graft with only heparin. However, the TEVGs in the LXW and CO&LXW groups were covered by the ECs, indicating almost complete endothelialization in the LXW-7 functionalized vascular grafts. The coverage ratio of ECs in the Control, LXW, and CO&LXW groups was (25.6 ± 11.8) %, (93.0 ± 1.0) %, and (92.3 ± 5.0) %, respectively (Fig. 7f).

The TEVGs were sliced and then examined by HE and immunofluorescence staining. It was obvious that the neointimal hyperplasia appeared in the Control group (Fig. 7g). The intimal thickness of the TEVGs in the CO&LXW group was approximately half of that in the Control group (Fig. 7h). Most of the cells on the luminal surface were CD31 positive in both the LXW and CO&LXW groups. Particularly, many CD31^+^&α-SMA^+^ cells were found to exist in the neointima of LXW, and the ECs lining in the vascular grafts appeared to be loosely arranged, suggesting that EndMT occurred within these vascular grafts (Fig. 7i). In contrast, hardly any CD31^+^&α-SMA^+^ cells could be observed in the CO&LXW group, where the endothelium was intact and the ECs formed a tightly connected monolayer. However, in the Control group, nearly all of the cells in the neointima expressed α-SMA, but very few CD31^+^ cells were observed. Interestingly, these cells might be derived from macrophages, as evidenced by the high expression of F4/80 (Fig. 7j). Furthermore, the neointima in the LXW group was invaded by the F4/80^+^ cells due to its loosely arranged endothelium, while very few F4/80^+^ cells could be seen in the CO&LXW group. The pro-inflammatory cytokines TNF-α and IL-1β were found to accumulate in the neointima of the Control and LXW groups but were negligible in the CO&LXW group (Fig. 7k).

**Fig. 8:**
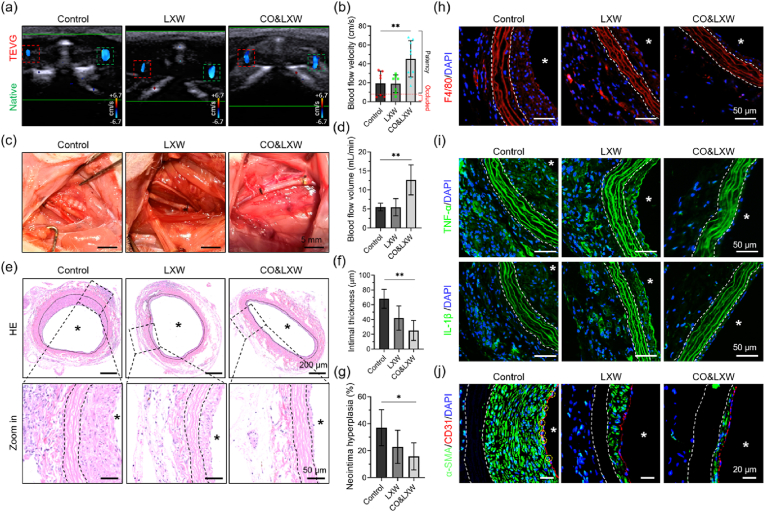
Characterization of the TEVGs after implantation for 21 and 28 days in the carotid arteries of SD rats. (a) Representative ultrasound images of the TEVGs after implantation for 21 days. (b) Blood flow velocity in the vascular grafts after 21 days. (c) Macroscopic views of the TEVGs after implantation for 28 days. (d) Statistical analysis of the blood flow volume in the vascular grafts after 28 days. (e) HE images of the TEVGs after implantation for 28 days. (f) Thickness and (g) hyperplasia of the neointima as measured from the HE images. Immunofluorescence staining of F4/80 (red) (h), TNF-α (green), and IL-1β (green) (i), and the associated CD31 (red) and α-SMA (green) (j) in the implanted TEVGs, with cell nuclei stained with DAPI. The lumen of the TEVGs is indicated with asterisks. Data are presented as means ± SD (n ≥ 3) and analyzed using one–way ANOVA, *p < 0.05, **p < 0.01, ***p < 0.001, *ns*: not significant.

After 3 weeks of implantation, the TEVGs in both the Control and LXW groups were found to have marked intimal hyperplasia, as shown in the wave spectrograms and the color Doppler ultrasound images (Fig. S12). Meanwhile, the lumen area of the vascular grafts was much smaller than that of the native blood vessels in the Control and LXW groups, while no significant difference was observed in the CO&LXW group (Fig. 8a). The blood flow velocity in the vascular grafts was also measured, and the cases with cuff fell off or host-vascular thrombus were excluded in statistical analysis (Fig. 8b). The blood flow velocity in the CO&LXW group was 45.4 ± 19.2 cm/s, whereas it decreased to 19.3 ± 13.1 cm/s and 19.0 ± 9.5 cm/s in the Control and LXW groups, respectively. Three out of seven vascular grafts in the Control group exhibited extremely low blood flow (<10 cm/s) and became occluded after 4 weeks of implantation. Similarly, one of the TEVGs in the LXW group (*n* = 7) experienced the same outcome. However, all vascular grafts in the CO&LXW group (*n* = 9) remained patent during the implantation period.

In the macroscopic photographs, the CO&LXW vascular grafts appeared to be filled better than those in the other two groups, and some yellow CO@COF nanoparticles were still observed on these TEVGs after 4 weeks of transplantation (Fig. 8c). The blood flow volume was calculated as 5.5 ± 1.0 mL/min, 5.5 ± 2.3 mL/min, and 12.7 ± 3.9 mL/min in the Control, LXW, and CO&LXW groups, respectively (Fig. 8d). The HE staining of the TEVGs showed obvious stenosis in both the Control and the LXW groups, while no noticeable intimal hyperplasia occurred in the CO&LXW group (Fig. 8e). The neointima thickness and degree of hyperplasia in the vascular grafts were measured using ImageJ software. The results revealed a significant reduction in both indicators in the LXW group compared with the Control group. Notably, when CO nanogenerators were present, a greater decline was observed in these parameters (Fig. 8f and g). Many F4/80^+^ cells were found to be located in the extima of the vascular grafts in both the Control and the LXW groups, which could be unequivocally identified as macrophages. Furthermore, hardly any macrophage infiltration was observed within the neointima of all these TEVGs (Fig. 8h). The distribution of TNF-α and IL-1β was quite similar to that in the macrophages (Fig. 8i). Cells lying between the vascular media and the endothelium expressed α-SMA but without F4/80, which was different from that on day 14. Hence, these cells could be considered as the mature SMCs. It was obvious that the luminal surface of all the TEVGs was covered with CD31^+^ cells. The only difference was that most of the CD31^+^ cells expressed α-SMA in the Control group, suggesting that these cells were undergoing EndMT (Fig. 8j and Fig. S13). These results indicated that the remolding of the vascular grafts in the LXW and CO&LXW groups reached stabilization, but the neointima in the Control group showed a trend of continuous growth in the future.

**Fig. 9:**
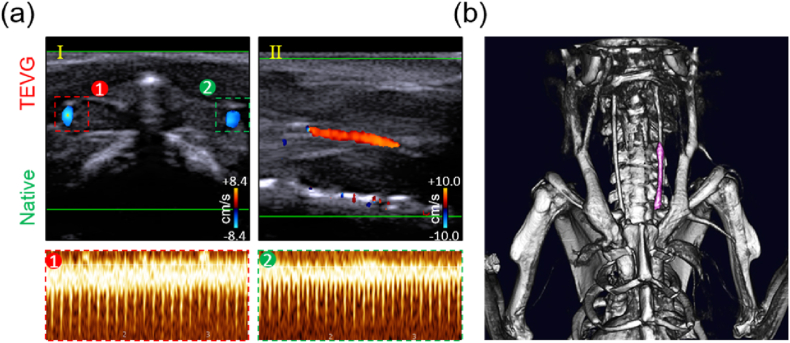
Patency of the CO@COF vascular grafts 3 months post-implantation. (a) Doppler ultrasound images of the native blood vessel and the implanted vascular grafts in the carotid artery of SD rats. (b) Micro-CT analysis of the SD rat with TEVG (purple) transplantation. Iohexol was injected as the contrast medium.

The TEVGs from the CO&LXW group were left in the SD rats for 3 months, and their patency was confirmed through Doppler ultrasound and micro-CT analysis. The results showed that the TEVGs had a steady blood flow, and their lumen area showed nearly no difference compared with the native blood vessel on the right side (Fig. 9a). The ultrasound chart plotted under the pulse wave model revealed that these TEVGs had clear pulsation, which was quite similar to that of the native blood vessel. In addition, no obvious aneurysms, intimal hyperplasia, and thrombus were observed by micro-CT analysis (Fig. 9b).

## 4 Discussion

Small-diameter TEVGs have shown significant prospects for coronary artery bypass grafting, vascular defect repair, and tissue construction. However, some problems still exist in engineering TEVGs with long-term patency, especially the thrombosis and intimal hyperplasia [57]. To address these issues, numerous strategies have been developed to prevent blood clot formation, enhance endothelialization, or inhibit neointimal hyperplasia within the vascular grafts [[58], [59], [60]]. However, most of approaches exhibit limited functionality and hardly to simultaneously fulfill the requirements for anticoagulation and anti-stenosis. The integration of such intricate beneficial characteristics into one TEVG is very challenging. In this study, TEVGs were constructed by immobilizing CO nanogenerator, heparin, and LXW-7 peptide onto the acellular blood vessels. This created a multifunctional surface on the grafts that could effectively mitigate thrombosis, promote endothelialization and inflammatory resolution, and inhibit EndMT-mediated intimal hyperplasia, thereby significantly enhancing the patency of these small-diameter TEVGs.

In the previous study, we found that the TEVGs with weak anticoagulation properties were susceptible to thrombosis, leading to rapid blockage post-transplantation [16]. Therefore, it is crucial to prevent thrombus formation in the early stage before endothelization. Although anticoagulant drugs can be beneficial in preventing thrombosis, they also pose risks such as bleeding. Heparin has been demonstrated to effectively prevent thrombosis when coated onto intravascular implants [61,62]. In this study, covalent immobilization of heparin was used to create an anticoagulant surface on TEVGs. Very few platelets were found to adhere or be activated on the TEVGs, as demonstrated by *in vitro* platelet adhesion tests. Additionally, no noticeable thrombus formation was observed in whole-blood tests. Short-term and long-term observations in animal models revealed that none of the vascular grafts were blocked by thrombus formation. However, TEVGs coated only with heparin exhibited delayed endothelialization and significant intimal hyperplasia. This was primarily due to an extensive inflammatory response, resulting in blockages in some of the TEVGs and compromising their long-term patency.

The endothelium is in direct contact with the blood and acts as a physical barrier in regulating thrombosis, inflammatory response, and proliferation of SMCs. Therefore, endothelialization is essential for maintaining the long-term therapeutic efficiency of the implanted blood contact devices. Endothelium formation is closely related to the migration and proliferation of ECs, as well as the homing, differentiation, and proliferation of EPCs. It can be promoted by immobilizing the bioactive molecules that can enhance the corresponding physiological processes [63,64]. In this study, the LXW-7 peptide was immobilized onto the TEVGs using bioorthogonal chemistry, and this approach effectively promoted the endothelialization of the TEVGs. LXW-7 is a cyclic peptide derived from αvβ3 integrin ligand GRGD that has a strong binding affinity for primary EPCs/ECs [65]. The results demonstrated that immobilizing LXW-7 could significantly promote the adhesion of ECs/EPCs. A large number of ECs/EPCs were found to adhere to LXW-7-functionalized TEVGs after 24 h of transplantation, and these grafts showed complete endothelialization on the neointima after 14 days. In contrast, the cells in the Control group without LXW-7 did not achieve endothelialization at this time, and displayed severe intimal hyperplasia.

The inflammatory response induced by the implantation of TEVGs plays a crucial role in regulating graft remodeling. On the one hand, the inflammatory cells may directly lead to stenosis in TEVGs. Our results indicated that a large number of cells expressing F4/80 and α-SMA were present in the neointima of the heparin-functionalized TEVGs after 14 days of transplantation. After 28 days of transplantation, fewer F4/80 positive cells were observed in the neointima of these TEVGs, while most of the cells expressed α-SMA.It indicated that a potential transformation of adherent macrophages into SMC-like cells, thereby contributing to the development of intimal hyperplasia. This interesting phenomenon has caught our attention and more experiments are being conducted in our group to verify the origin of these cells. In addition, the immobilization of LXW-7 effectively reduced the attachment of platelets and WBCs *in vivo*, lowering the risk of thrombosis and inflammation in our TEVGs. On the other hand, the release of inflammatory factors can regulate the biological behavior of SMCs/ECs/EPCs, thereby affecting the patency of the TEVGs. Bioactive molecules secreted by macrophages and platelets, such as TGF-β, TNF-α, and ROS, can drive the phenotypic transformation of the anastomotic SMCs from a resting state to a synthetic phenotype, enhancing the migration, proliferation, and ECM secretion of the SMCs [66]. These factors can also trigger EndMT in the neo-endothelium [67,68]. Therefore, the inflammation should be quickly resolved to maintain the normal physiological function of the neo-endothelium to prevent pathological remodeling in the TEVGs. In our TEVGs, the CO nanogenerator could effectively remove ROS while releasing CO, inhibiting the ROS-induced apoptosis and EndMT in the homing ECs/EPCs. Furthermore, the CO nanogenerator inhabited the activation of inflammatory cells, reduced the secretion of pro-inflammatory factors, and promoted the transformation of inflammatory cells from a pro-inflammatory phenotype M1 to an anti-inflammatory phenotype M2.

As the resolution of inflammation, the neo-endothelium preserved its biological functions, thereby mitigating EndMT-mediated pathological remodeling in the TEVGs. From our results, many ECs/EPCs were found to be captured on the LXW-7-functionalized TEVGs after 14 days of implantation, as evidenced by the CD31 expression. However, part of the ECs expressed α-SMA, suggesting that these cells were undergoing EndMT. Due to the loss of intercellular connections, some F4/80^+^ cells infiltrated into the lower layer of the endothelium. In contrast, no similar phenomenon was observed in the CO&LXW group which had a significantly lower inflammation response. EndMT also led to more severe intimal hyperplasia in the LXW group than in the CO&LXW group after 28 days. It should be noted that when ECs are exposed to transient stimuli, the EndMT process can be reversible [69]. And the neointimal hyperplasia will stabilize due to endothelialization after 1–2 months, no longer causing significant graft stenosis [70]. Our results indicated that the endothelium returned to normal after 28 days following implantation in the LXW group, with no obvious inflammation detected. In contrast, although complete endothelialization was observed in the Control group, many ECs were still undergoing EndMT. As a result, the intima in the Control group might grow continuously in the future. These results demonstrated the importance of promoting endothelialization and maintaining the biological functions of the neo-endothelium in improving the patency of TEVGs.

## 5 Conclusions

In summary, we have successfully integrated heparin, LXW-7 peptide, and CO nanogenerator onto the acellular blood vessels to develop TEVGs with a multifunctional surface. This approach can effectively prevent thrombosis, promote endothelialization and inflammatory resolution, and inhibit intimal hyperplasia in the TEVGs. Our TEVGs have achieved long-term patency in animal model and show great potential in various biomedical applications. More importantly, this study has demonstrated that maintaining the health of the neo-endothelium is crucial to prevent pathological remolding in the TEVGs, which provides new clues for the design of vascular implants.

## 6 Data availability

The raw/processed data required to reproduce these findings are available from the authors.

## Ethics approval and consent to participate

All animal experiments in this study were performed following the approval from the Laboratory Animal Welfare and Ethics Committee of the Third Military Medical University (No. AMUWEC2020039). And the animals were obtained from the Experimental Animal Center of the Third Military Medical University.

## Conflict of interest

The authors declare that they have no known competing financial interests or personal relationships that could have appeared to influence the work reported in this paper.

## CRediT authorship contribution statement

**Yonghong Fan:** Writing – original draft, Visualization, Software, Methodology, Investigation, Funding acquisition, Data curation, Conceptualization. **Juan Pei:** Software, Methodology, Formal analysis, Data curation. **Yinhua Qin:** Methodology, Investigation, Formal analysis, Data curation. **Huifang Du:** Methodology, Investigation, Data curation. **Xiaohang Qu:** Software, Methodology, Investigation. **Wenya Li:** Methodology, Investigation, Data curation. **Boyue Huang:** Software, Methodology, Investigation. **Ju Tan:** Resources. **Yong Liu:** Resources, Methodology. **Gang Li:** Resources. **Ming Ke:** Writing – review & editing, Methodology, Conceptualization. **Youqian Xu:** Writing – review & editing, Software, Funding acquisition, Formal analysis. **Chuhong Zhu:** Writing – review & editing, Supervision, Project administration, Funding acquisition, Conceptualization.
