# Supplementary material for: Construction of tissue-engineered vascular grafts with enhanced patency by integrating heparin, cell-adhesive peptide, and carbon monoxide nanogenerators into acellular blood vessels
Source: Bioact Mater. 2023 Dec 28;34:221–36. doi: 10.1016/j.bioactmat.2023.12.015 (PMC10792202; doi:10.1016/j.bioactmat.2023.12.015)
Supplement: Multimedia component 1 [file mmc1.docx]

**Supplementary tables and figures**

**Table S1** The sequences of the primers in the RT-qPCR experiments.

| Primers | Sequence (5'-3') |
| --- | --- |
| CD31 | F- TCGTGGTCAACATAACAGAACT |
|  | R- TTGTACCATTCTTCTGCCTC |
| Vimentin | F-GCAAAGATTCCACTTTGCGT |
|  | R-GAAATTGCAGGAGGAGATGC |
| Fibronectin | F-AAACCAATTCTTGGAGCAGG |
|  | R-CCATAAAGGGCAACCAAGAG |
| TGF-βR1 | F-ACGGCGTTACAGTGTTTCTG |
|  | R-GCACATACAAACGGCCTATCT |
| Smad2 | F-GCCATCACCACTCAAAACTGT |
|  | R-GCCTGTTGTATCCCACTGATCT |
| CD144 | F-TCTGGTTGTCACAGGTGGAA |
|  | R- CTTGCGACTCACGCTTGACT |
| VEGF | F-GAGGAGCAGTTACGGTCTGTG |
|  | R-TCCTTTCCTTAGCTGACACTTGT |
| β-actin | F-ATCAAGATCATTGCTCCTCCTGAG |
|  | R-CTGCTTGCTGATCCACATCTG |
| SM-Calponin | F-CTGGCTGCAGCTTATTGATG |
|  | R-CTGAGAGAGTGGATCGAGGG |
| α-SMA | F-CAAAGCCGGCCTTACAGAG |
|  | R-AGCCCAGCCAAGCACTG |


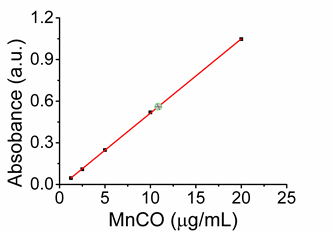


**Figure S1** The standard curve of MnCO used in this study.


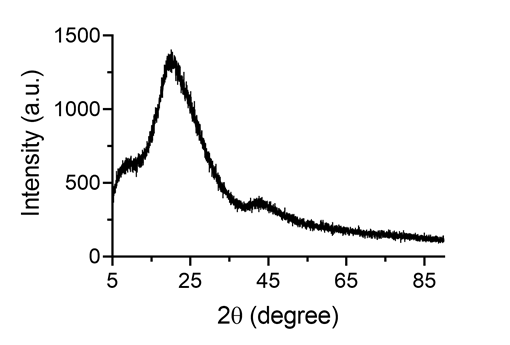


**Figure S2** XRD pattern of the nano COFs.


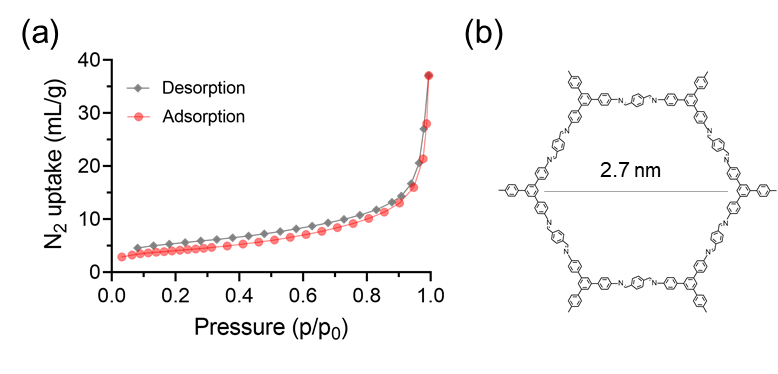


**Figure S3** (a) N_2_ adsorption–desorption isotherms of the COFs nanoparticles, and (b) the pore size as calculated by BJH analysis.


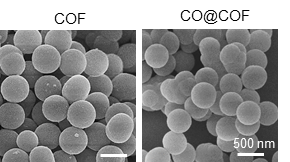


**Figure S4** SEM observation of the COFs and CO@COF nanoparticles.


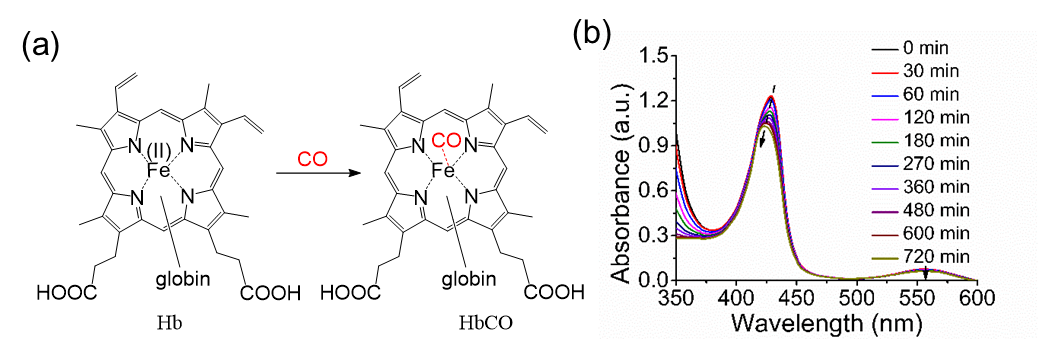


**Figure S5** (a) Detecting CO release by Hb method. (b) Monitoring of CO release from the CO@COF in the PBS under 500 μM H_2_O_2_ with Hb.


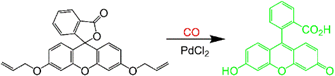


**Figure S6** Fluorescent detection of CO by CO probe and PdCl_2_.


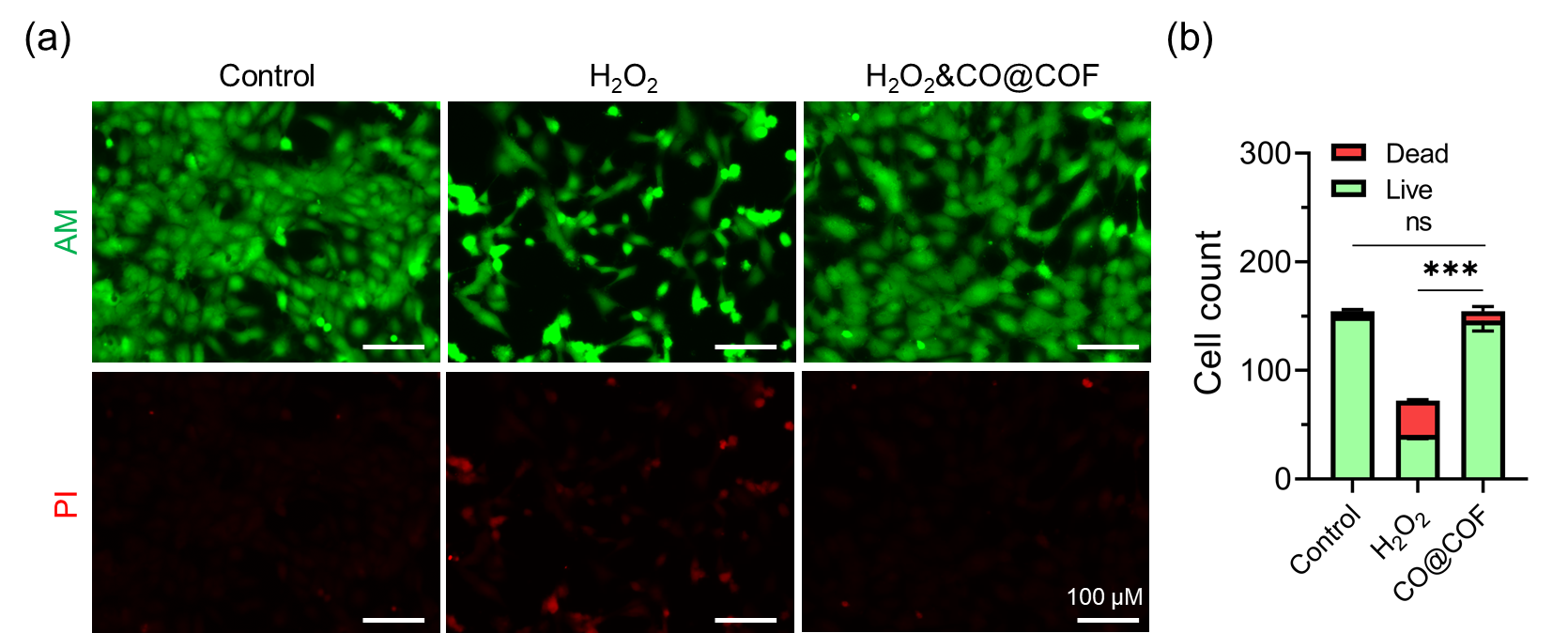


**Figure S7** (a) Apoptosis in HUVECs under 100 μM H_2_O_2_ stimulation as visualized by LVE/DEAD staining kit. (b) Statistical analysis of cell death according to the fluorescent images.


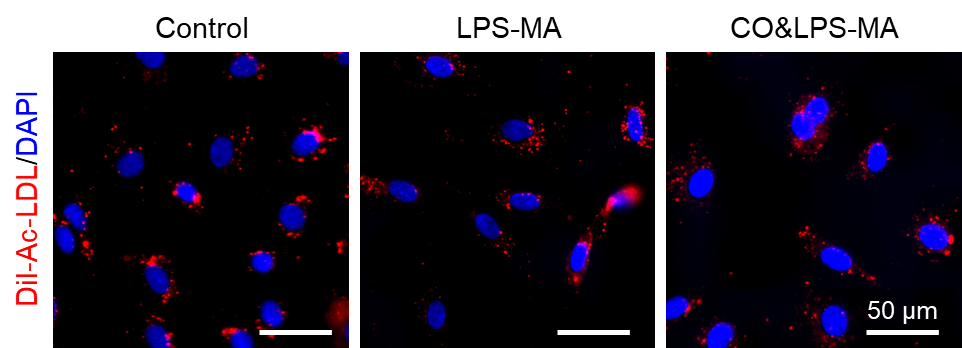


**Figure S8** Uptake of Dil-Ac-LDL of the HUVECs after treated with LPS activated macrophages.

**
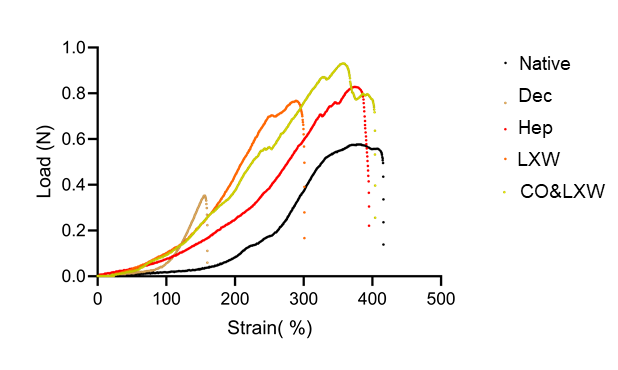
**

**Figure S9** Tensile-strength of the TEVGs. Native-native blood vessel, Dec-decellularized blood vessel, Hep-TEVG with heparin immobilization, LXW-TEVG with heparin and LXW-7 immobilization, CO&LXW-TEVG with heparin, LXW-7 and CO@COF immobilization


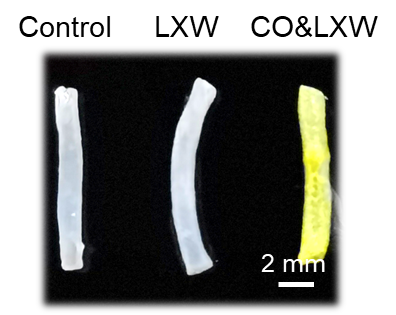


**Figure S10** Macroscopic view of the TEVGs.


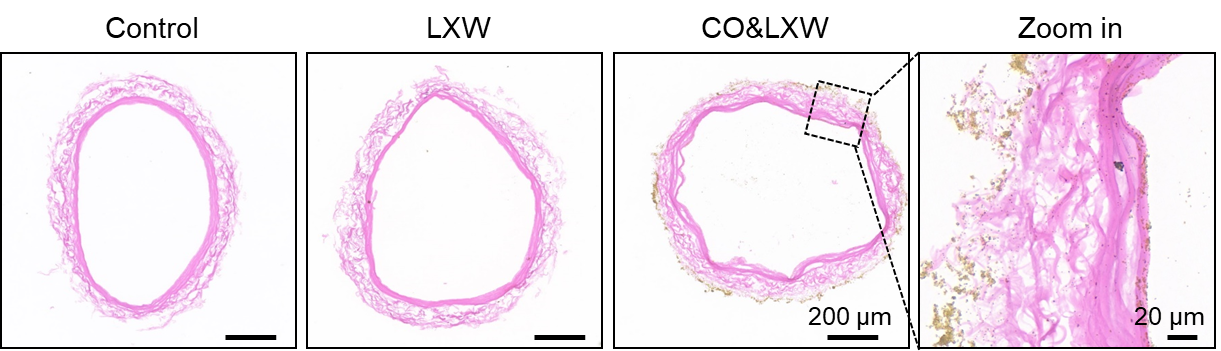


**Figure S11** HE staining of the TEVGs.


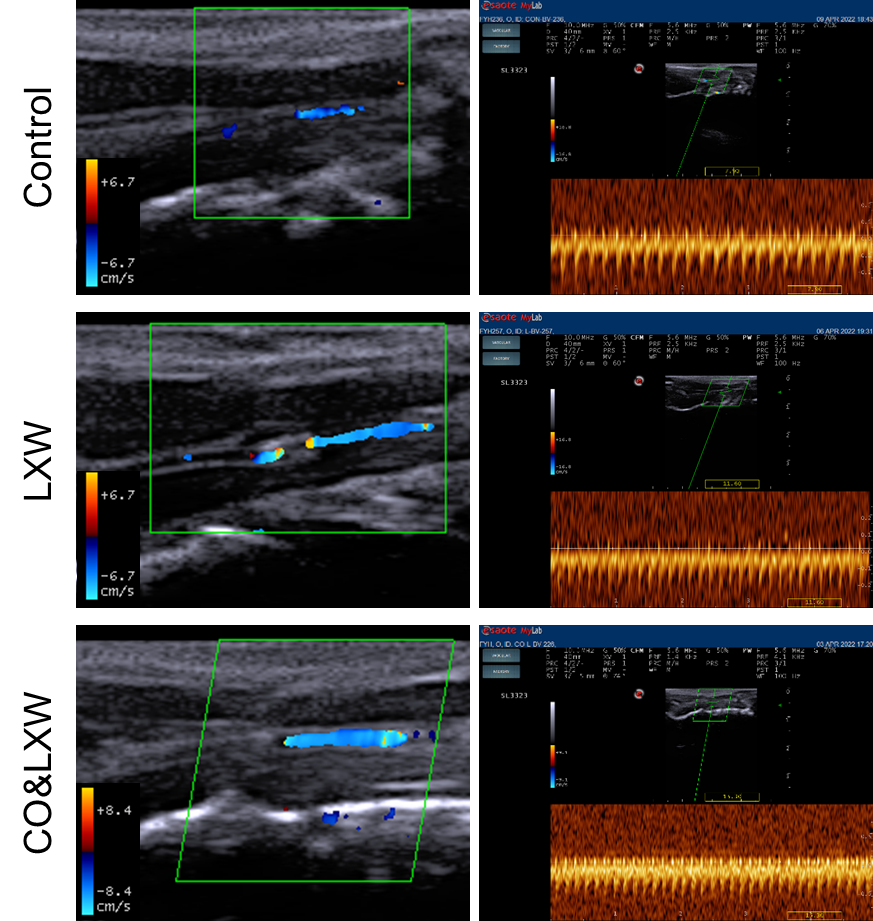


**Figure S12** Doppler ultrasound image of the TEVGs after 3 weeks of implantation.


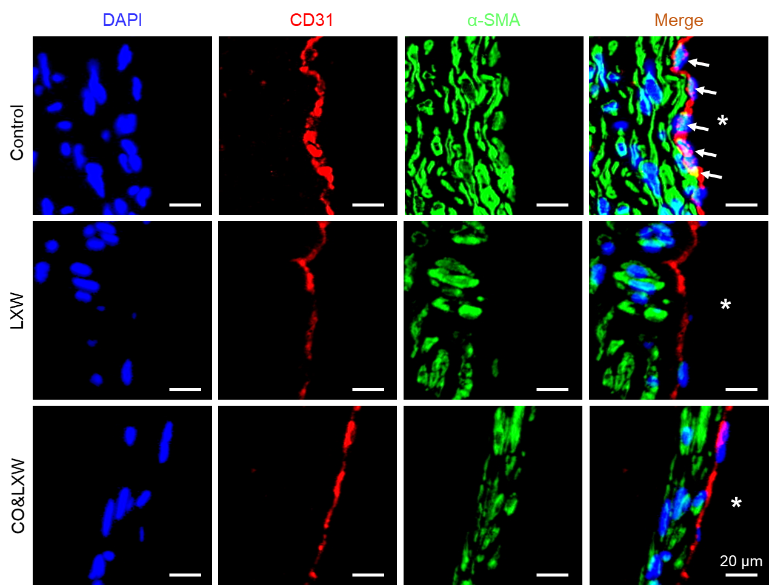


**Figure S13** Immunofluorescence staining of CD31 (red) and α-SMA (green) in the TEVGs after implantation for 28 d, the cell nuclei were stained with DAPI.
